# Genetic gain for rice yield in rainfed environments in India

**DOI:** 10.1016/j.fcr.2020.107977

**Published:** 2021-01-01

**Authors:** Arvind Kumar, Anitha Raman, Shailesh Yadav, S.B. Verulkar, N.P. Mandal, O.N. Singh, P. Swain, T. Ram, Jyothi Badri, J.L. Dwivedi, S.P. Das, S.K. Singh, S.P. Singh, Santosh Kumar, Abhinav Jain, R. Chandrababu, S. Robin, H.E. Shashidhar, S. Hittalmani, P. Satyanarayana, Challa Venkateshwarlu, Janaki Ramayya, Shilpa Naik, Swati Nayak, Manzoor H. Dar, S.M. Hossain, Amelia Henry, H.P. Piepho

**Affiliations:** a International Rice Research Institute (IRRI), DAPO Box 7777, Metro Manila, Philippines; b IRRI South Asia Regional Center (ISARC), Varanasi, India; c Indira Gandhi Krishi Vishwavidyalaya (IGKV), Raipur, India; d Central Rainfed Upland Rice Research Station (CRURRS), ICAR-NRRI, Hazaribagh, India; e National Rice Research Institute (NRRI), Cuttack, India; f Indian Institute of Rice Research (IIRR), Hyderabad, India; g Narendra Dev University of Agriculture and Technology (NDUAT), Ayodhya, India; h ICAR Research Complex for NEH Region, Tripura Centre, Lembucherra, India; i Banaras Hindu University (BHU), Varanasi, India; j Bihar Agricultural University (BAU), Sabour, India; k ICAR-Research Complex for Eastern Region, Patna, India; l Barwale Foundation, Hyderabad, India; m Tamil Nadu Agricultural University (TNAU), Coimbatore, India; n University of Agricultural Sciences (UAS), Bangalore, India; o Regional Rice Research Station, Maruteru, Acharya NG Ranga Agricultural University, (ANGRAU), Guntur, India; p International Rice Research Institute, South Asia Hub, ICRISAT, Patancheru, Hyderabad, India; q International Rice Research Institute (IRRI), New Delhi, India; r International Rice Research Institute (IRRI), Bhubaneshwar, India; s Universitaet Hohenheim, Biostatistics Unit, 70593, Stuttgart, Germany

**Keywords:** Rice, Drought, Genetic gain, Yield potential

## Abstract

The complexity of genotype × environment interactions under drought reduces heritability, which determines the effectiveness of selection for drought tolerance and development of drought tolerant varieties. Genetic progress measured through changes in yield performance over time is important in determining the efficiency of breeding programmes in which test cultivars are replaced each year on the assumption that the new cultivars will surpass the older cultivars. The goal of our study was to determine the annual rate of genetic gain for rice grain yield in a drought-prone rainfed system in a series of multi-environment trials conducted from 2005 to 2014 under the Drought Breeding Network of Indian sites in collaboration with the International Rice Research Institute (IRRI). Our results show a positive trend in grain yield with an annual genetic yield increase of about 0.68 % under irrigated control, 0.87 % under moderate reproductive stage drought stress and 1.9 % under severe reproductive stage drought stress due to breeding efforts. The study also demonstrates the effectiveness of direct selection for grain yield under both irrigated control as well as managed drought stress screening to improve yield in typical rainfed systems. IRRI's drought breeding programme has exhibited a significant positive trend in genetic gain for grain yield over the years under both drought stress as well as favorable irrigated control conditions. Several drought tolerant varieties released from the programme have outperformed the currently grown varieties under varied conditions in the rainfed environments on farmers’ fields.

## Highlights

• Genetic gain for rice grain yield for International Rice Research Institute drought breeding program was estimated.
• Positive trend of 0.68 %, 0.87 %, 1.9 % under irrigated control, moderate and severe drought achieved.
• Superiority of new rice varieties over currently grown demonstrated on farmers’ fields.
• International Rice Research Institute developed rice varieties can protect farmers from crop losses under drought conditions.

## 1 Introduction

Rice is the most important crop in the world feeding more people than any other crop, and plays a vital role in the Asian economy. In 1966, the release of IR8, the first semi-dwarf high yielding modern rice variety from a cross between Peta, a tall Indonesian variety, and Dee-geo-woo-gen, a short statured variety from Taiwan, marked the initiation of the green revolution in rice production that over time has transformed the food deficient Asia to a food self-sufficient region. The green revolution had a remarkable impact on rice production, following which rice farming underwent significant transformation. The improved short-statured varieties (such as Jaya, IR20, IR36, IR42, IR50) developed since then for the irrigated ecosystem had high yield potential, short growth duration, were input responsive, and were disease and insect resistant, however they lacked improved grain quality (Khush, 1987; Khush and Virk, 2005). IR64, released in the Philippines in 1985 had superior grain quality as well as higher yield compared with previously released IR varieties, and its long persistence in farmers’ fields after its release was attributed to the excellent eating quality (Champagne et al., 2010; Mackill and Khush, 2018). The green revolution was based upon the philosophy that selection can be done under optimal environments assuming that an increased yield potential will have a carryover effect under water shortage conditions. However, in the regions chronically affected by drought, that approach failed to improve yield under drought conditions (Ceccarelli, 2010). Thus, by focusing on technologies for very favorable, usually irrigated environments, millions of hectares of land in rainfed areas were not able to fully benefit from yield gains made in irrigated areas (Ziegler, 1998).

Rice production systems can be classified into lowland and upland. Rainfed rice fields that are not irrigated but in which the soil is flooded for at least part of the crop cycle are commonly known as rainfed lowlands. Water availability is unpredictable, as the crop is rainfed. Asia has about 46 million hectares of rainfed lowland rice or almost 30 % of the total world rice area (Maclean et al., 2002). One-third of South and South East Asian rice lands lie within this ecosystem, which dominates rice areas of Bangladesh, Cambodia, Myanmar, Nepal, and Thailand, and is important in India, Indonesia, Laos, and Vietnam (CGIAR, 1998). More than 75 % of the region’s poor rice farmers depend on rainfed agriculture. The uncertain water supply in rainfed lowland areas, together with the infertile soil that can be acidic or saline and the varying crop management methodologies, provide a highly heterogeneous set of breeding targets with a range of environmental conditions that influence the phenotypic response of the genotypes. This complex and diverse situation very often lies within small geographical regions. Genetic improvement under such complex situations would be a key challenge for any plant breeding program.

In the rainfed environments characterized by lack of water control, drought and flooding are regular problems. Drought is the most significant constraint affecting rice production in rainfed lowland rice. The intensity, duration and timing of drought may vary from location to location and in a given location from year to year. At reproductive stage, drought causes a reduction in the number of grains per panicle, increases grain sterility, and reduces grain weight (Pantuwan et al., 2002). Drought during the reproductive stage leads to major yield reduction (Kumar et al., 2007). Even moderate drought stress at reproductive stage can result in substantial reduction in grain yield (O’Toole, 1982; Huke and Huke, 1997; Zhang et al., 2018). Moreover, drought seldom occurs in isolation; it often interacts with other abiotic and biotic stresses (Ceccarelli and Grando, 1996) such as soil texture, pH, soil fertility, diseases and insects. With the effect of climate change, the recurrence and intensity of drought is likely to change (Ceccarelli et al., 2004) and possibly becomes more frequent (World Bank, 2009). Recurrent drought linked with climate change would have an adverse effect on crop productivity and on millions of poor farmers’ livelihood. Therefore, drought-prone rice systems require stress tolerant rice varieties and improved management strategies.

However, breeding for drought resistance has been slow. In addition to drought tolerance being a complex trait, it is reported to be controlled by several genes each with small effects, and significant genotype × environment interactions (G × E) under stress (Dixit et al., 2014) lead to variable performance of genotypes across locations. Consequently, yield under drought has been reported to have low heritability (H) which complicates selection of superior, drought tolerant, stable genotypes. In turn, this limits genetic gain for grain yield under drought. Genetic gain, although a less commonly used measure of breeding efficiency (Ceccarelli, 2015), is defined as the amount of increase in performance that is achieved annually through artificial selection (Xu et al., 2017). Genetic gain estimation is vital for any crop breeding program to analyze its strengths and weaknesses and to plan future breeding activities. In stress environments, improving selection efficiency and increasing genetic gain requires a proper understanding of the target environment, a selection environment that is representative of the target environment, a population with large genetic variance, proper trial management, and appropriate screening procedures.

Genetic gain is usually estimated using multi-environment trials (METs) that are routinely conducted as a part of breeding program (Muralidharan et al., 2002; Fufa et al., 2005). Traditionally, popular check cultivars are grown in all trials in METs to minimize the influences of location and year. Genetic progress has been estimated from the difference between checks and top-yielding cultivars (Feyerherm et al., 1984) or by subtracting the trend line fitted to a set of checks common across many years from the trend line fitted to the year-wise means (Sharma et al., 2012). However, choice of a representative check cultivar becomes complicated and with the introduction of new crop genotypes, the check cultivar may become unrepresentative and a new check cultivar has to be chosen (Friesen et al., 2016). In previous analyses, the genotype yields were often expressed as a percentage of the long-term check yield, assuming that this would minimize G × E, which may not be realistic. Moreover, data generated from METs aimed at screening of new cultivars are highly unbalanced due to variation in the genotypes tested from year to year, variation in the number of replicates at each site and variation in the sites that are included from year to year. In addition, stress-susceptible varieties may contribute to missing values when exposed to severe stress (Raman et al., 2012). Therefore, the analytical methods for METs need to be adaptable to unbalanced data (Liu et al., 2015). Mixed model analysis is an alternative to the traditional MET analysis based on checks. Missing values, parameter estimation and prediction of genotype performances are effectively handled by mixed models (Piepho and Möhring, 2006). Treating genotypes as random effect in a mixed model allows their genetic value to be predicted using the best linear unbiased predictors (BLUPs). Recently, mixed models have been used to dissect genetic and non-genetic trends in multi-environment trial data (Mackay et al., 2011; Piepho et al., 2017; Laidig et al., 2014, 2017; Ahrends et al., 2018). The contribution of the cultivars (genetic trend) and of the environments (non-genetic trends) to yield improvement over time is quantified by regression coefficients.

The genotypes screened in this study are breeding lines developed from crosses involving a high yielding, but drought susceptible recipient parent that possesses good grain quality, and a low yielding but highly drought-tolerant donor parent. The lines were advanced through direct selection for grain yield (Kumar et al., 2007) under both favorable conditions as well as variable levels of reproductive-stage drought stress in different generations in a pedigree generation advancement breeding program. The selected lines were evaluated for their performance for grain yield under favorable irrigated condition as well as moderate and severe levels of reproductive stage drought stresses in the years 2005–2014 (Verulkar et al., 2010; Mandal et al., 2010). The experiments were conducted in the Indian sites in collaboration with the International Rice Research Institute (IRRI) under the Drought Breeding Network of the STRASA (Stress-Tolerant Rice for Africa and South Asia) project. The STRASA project, supported by the Bill and Melinda Gates Foundation, has helped millions of farmers who produce their crop under predominantly rainfed conditions to achieve significantly higher yield despite abiotic stresses such as drought, flood, cold, salinity and iron toxicity (Ismail et al., 2013; Dar et al., 2014; Arora et al., 2019).

The objective of the present study under STRASA was to assess and monitor yield trends and to estimate the annual rate of increase in yield for breeding lines in the highly variable drought prone rainfed lowland environments of eastern and southern India. The trends were dissected to quantify the contributions of varietal selection and crop management to the improvement in grain yield. Comparisons draw on yield performance and/or tolerance to different biotic/abiotic stresses, of the released stress tolerant rice varieties (STRVs) as well as the popular farmer varieties.

## 2 Material and methods

### 2.1 Field sites, genotypes and years of screen

The data comprise yield measurements from rainfed lowland trials conducted under three pre-defined conditions of water and agronomic management; 1. Irrigated control (maintaining the plants under well-watered conditions until physiological maturity) conditions, 2. rainfed with supplementary irrigation and, 3. rainfed conditions.

Approximately 50–70 selected genotypes of 100–120 days duration were pooled to constitute an advanced yield trial (AYT). These AYT were conducted during wet seasons for ten years from 2005 to 2014 at fourteen on station test sites in India (Table 1,2).

**Table 1:** Salient features of the STRASA network sites.

Sites	Location	Target environment	Soil type	Field topography	Drought	Presently grown varieties
Indira Gandhi Krishi Vishwavidyalaya (IGKV), Raipur, Chhattisgarh	21° 14′ N, 81° 38′ E	Rainfed low land ecosystem	Clay, clay loam, low organic carbon	Bunded, shallow lowland to mid lowland	Reproductive stage and early season drought	MTU 1010, IR 64, Mahamaya, Swarna
Narendra Dev University of Agriculture and Technology (NDUAT), Faizabad, Uttar Pradesh	26° 47′ N, 82° 12′ E	Rainfed shallow, mid and deep low land	Clay, clay loam, low organic carbon	Bunded, shallow, mid and deep lowland	Early season drought and reproductive stage	Sarjoo 52, Swarna, NDR 97, NDR 359, Baranideep
Central Rainfed Upland Rice Research Station (CRURRS), Hazaribag, Jharkhand	23° 59′ N, 82° 25′ E	Bunded uplands and rainfed shallow low land	Acidic in nature, very poor in fertility, low in available N and organic carbon	Highly undulating, unbunded, bunded uplands and bunded shallow lowland	Drought at all stages of growth	IR 36, IR 64, MTU 1010, Hazaridhan, Sadabahar, Birsa 201, Some other land races
Central Rice Research Institute (CRRI), Cuttack, Odisha	20° 28′ N, 85° 54′ E	Rainfed upland and shallow lowland in dry season	Sandy loam and clay loam soil	Bunded, shallow, mid and deep lowlands	Seedling, and vegetative stage	Lalat, Swarna, Varshadhan, Naveen, MTU 1010, Khandagiri, Vandana,
TamilNadu Agricultural University (TNAU), Coimbatore, TamilNadu	11° 00′ N, 77° 00′ E	Irrigated lowland	Clay	Bunded, shallow and mid lowlands	Reproductive stage	IR 64, Co. 47
Tamil Nadu Agricultural University (TNAU), Paramakudi, TamilNadu	09° 31′ N, 78° 39′ E	Rainfed upland	Clay loam	Bunded Upland	Reproductive stage	PMK 3, ADT 38, Local races
University of Agricultural Sciences (UAS), Bangalore, Karnatakka	12° 58′ N, 77° 38′ E	Rainfed upland and shallow lowland	Red, loamy and light	Bunded, shallow lowland	Reproductive stage	MTU 1001, IR 64, Jyoti, MTU 1010, BPT 5204, Rasi,
Barwale Foundation (BF), Hyderabad, Telengana	17° 20′ N, 78° 30′ E	Rainfed Shallow low land	Clay loam	Bunded, shallow lowland	Reproductive stage	Sambha Mahsuri, Swarna, MTU 1010
Birsa Agricultural University (BAU), Ranchi, Jharkhand	23° 17′ N, 85° 19′ E	Rainfed bunded uplands and shallow low land	Acidic in nature, very poor in fertility, low in available N and organic carbon	Highly undulating, bunded uplands and shallow lowland	Drought at all stages of growth	Lalat, IR 36, IR 64, MTU 1010, Birsa 201, Some other land races

**Table 2:** Details of the Advanced Yield Trials (AYT) trials assigned to two stress levels based on the reduction in trial mean yield as compared to irrigated control during 2005 – 2014.

Irrigated control					Moderate stress					Severe Stress				
Trial	Location	year	Trial mean yield	Trial H	Trial	Location	Year	Trial mean yield	Trial H	Trial	Location	Year	Trial mean yield	Trial H
1	Faizabad	2005	6951	0.95	1	Raipur	2006	2796	0.94	1	Faizabad	2006	1265	0.94
2	Hazaribag	2005	4997	0.87	2	Faizabad	2007	2192	0.96	2	Hazaribag	2006	994	0.86
3	Raipur	2005	3814	0.87	3	Hazaribag	2007	3383	0.83	3	Raipur	2006	1223	0.91
4	Faizabad	2006	3658	0.98	4	Raipur	2007	3270	0.77	4	Raipur	2007	1889	0.8
5	Hazaribag	2006	2998	0.51	5	Barwale	2008	2013	0.92	5	TNAU	2007	1025	0.32
6	Raipur	2006	4750	0.61	6	Hazaribag	2008	2234	0.85	6	Barwale	2008	991	0.92
7	Barwale	2007	5480	0.48	7	Raipur	2008	2226	0.84	7	DRR	2008	1004	0.86
8	Faizabad	2007	4098	0.97	8	Faizabad	2009	2369	0.87	8	Faizabad	2008	1639	0.98
9	Hazaribag	2007	4567	0.72	9	Hazaribag	2009	2222	0.83	9	Hazaribag	2008	1605	0.91
10	Raipur	2007	3634	0.73	10	Raipur	2009	2470	0.68	10	Raipur	2008	1009	0.93
11	Barwale	2008	4108	0.84	11	Tripura	2009	2367	0.39	11	TNAU	2008	1126	0.74
12	Faizabad	2008	3183	0.99	12	Barwale	2010	2340	0.72	12	Barwale	2009	1178	0.96
13	Hazaribag	2008	5194	0.84	13	Raipur	2010	2708	0.67	13	Paramakudi	2009	1499	0.46
14	Raipur	2008	3929	0.84	14	TNAU	2010	3711	0.61	14	Raipur	2009	1895	0.7
15	Ranchi	2008	5268	0.73	15	Hazaribag	2011	2507	0.82	15	TNAU	2009	1935	0.98
16	Barwale	2009	3618	0.96	16	Patna	2011	2865	0.92	16	DRR	2010	833	0.96
17	Faizabad	2009	3940	0.97	17	Raipur	2011	2708	0.93	17	Ranchi	2010	1478	0.94
18	Hazaribag	2009	4475	0.93	18	Ranchi	2011	2482	0.7	18	TNAU	2010	1415	0.94
19	Raipur	2009	5258	0.68	19	Barwale	2012	3537	0.82	19	DRR	2011	1341	0.71
20	UAS	2009	4648	0.94	20	Patna	2012	2997	0.93	20	Raipur	2011	1786	0.69
21	Barwale	2010	3904	0.9	21	Tripura	2012	3514	0.98	21	Rewa	2011	1797	0.89
22	Faizabad	2010	3925	0.9	22	DRR	2013	3243	0.87	22	TNAU	2011	1635	0.65
23	Hazaribag	2010	4326	0.9	23	Faizabad	2013	3783	0.91	23	Hazaribag	2012	1815	0.94
24	Raipur	2010	5610	0.81	24	Patna	2013	3436	0.82	24	Rewa	2012	1714	0.59
25	Ranchi	2010	3405	0.97	25	Raipur	2013	3294	0.91	25	Hazaribag	2013	1537	0.66
26	TNAU	2010	4047	0.5	26	Hazaribag	2014	2953	0.89	26	Ranchi	2013	1850	0.55
27	DRR	2011	5371	0.45	27	Raipur	2014	2455	0.93	27	Sabour	2013	1438	0.97
28	Faizabad	2011	4101	0.96	28	Ranchi	2014	2523	0.74	28	TNAU	2013	528	0.97
29	Hazaribag	2011	5488	0.8						29	DRR	2014	958	0.9
30	Patna	2011	4093	0.93						30	Faizabad	2014	602	0.96
31	Raipur	2011	5185	0.78						31	Tripura	2014	1747	0.61
32	Barwale	2012	6035	0.57										
33	Faizabad	2012	4445	0.92										
34	Hazaribag	2012	5252	0.94										
35	Patna	2012	5918	0.94										
36	Raipur	2012	6183	0.82										
37	Sabour	2012	5135	0.77										
38	TNAU	2012	4609	0.73										
39	Tripura	2012	5620	0.7										
40	DRR	2013	5453	0.89										
41	Faizabad	2013	5061	0.87										
42	Hazaribag	2013	4804	0.74										
43	Patna	2013	5745	0.7										
44	Raipur	2013	5151	0.96										
45	TNAU	2013	4318	0.84										
46	DRR	2014	5645	0.69										
47	Faizabad	2014	4087	0.92										
48	Hazaribag	2014	6002	0.87										
49	IRRI-SA	2014	8336	0.36										
50	Maruteru	2014	4451	0.98										
51	Raipur	2014	6382	0.92										
52	Sabour	2014	4775	0.6										
53	Tripura	2014	5896	0.63										
54	Varanasi	2014	8284	0.79										

All trials were laid out in alpha-lattice designs with three replications. Wet-season trials followed a standardized drought-screening protocol at all sites (Verulkar et al., 2010; Kumar et al., 2014). In brief, the stress was imposed by draining out water from the field at 50 DAS (days after seeding) and the cyclic soil moisture deficit stress was maintained till the maturity stage. Perforated PVC pipes of 1.1 m length was installed at regular places across the field to measure depth of water table. A life-saving irrigation was provided as soon as the water table level in the PVC fell below 1.0 m from soil surface and all the susceptible checks started showing severe leaf rolling and dying at 10 am. Water was drained out immediately after 24 h to initiate the next cycle of stress.

The irrigated control trial was planted at each of the sites with similar crop management practices to determine grain yield reduction under stress. Standing water was maintained in the field of the irrigated control trials from transplanting until maturity. Under reproductive stage drought, the seeding and transplanting for stress trials was delayed by approximately 20–25 days to increase the probability of the reproductive stage of the crop to coincide with the end of monsoon rains. A detailed description for a list of typical planting delays at each site was given previously by Singh et al. (2017).

Most stress trials were conducted in light-soiled fields high in toposequence with the crop fully irrigated for one month after transplanting and then drained to ensure rapid drying. The rainfed condition trials were planted normally but were never irrigated, and unbunded fields were chosen to increase the probability of water stress. All three conditions were judiciously designed to reflect and simulate conditions of farmers’ fields.

To eliminate the bias of border effects, we created our field layout in such a way that the planted area was continuous, without leaving space between plots. The two hills (borders) neighboring the bund (edge of the field) were excluded from the harvest for yield assessment in all plots. All the hills in the plot area described above were harvested, and we discarded measurements made on plots with more than 20 % missing hills. A recommended agronomical practice previously reported by Kumar et al. (2014) was followed for irrigated control as well as drought trials conducted at IRRI, SA-Hyderabad. In brief, the row-to-row and plant-to-plant spacing was maintained at 20 cm × 20 cm. Nitrogen, phosphorus and potassium (NPK) were applied at the rate of 120:60:40 kg ha^−1^. N was applied in three doses, first as basal, second at tillering stage and third at panicle initiation while P and K were applied as basal in a single dose. In order to control weeds, Sofit (pretilachlor ± safene, 0.3 kg a.i. ha^−1^) was sprayed just after transplanting followed by Furadan (carbofuran 1 kg a.i. ha^−1^) and Dimotrin (cartap hydrochloride, 0.25 kg a.i. ha^−1^) at 5 and 16 days after transplanting, respectively. The detailed information on the trials conducted at various sites in India (location, year of conduct, harvested area, dose of fertilizers applied, rainfall during the reproductive-stage stress period) is presented in Supplementary Table S1.

The experimental sites included in this study represented the major drought-prone rice-growing regions in India. A description of the land-use history, soil and environmental characteristics of these sites, including the 2012–2014 AYT trials in the present study, was reported by Singh et al. (2017).

After harvest, each stress trial was classified as moderately stressed if the yield reduction as compared to the irrigated control trial was 30–65 %, and severely drought-stressed if the yield reduction was more than 65 % (Table 2). Trials with less than 30 % yield reduction were classified as mild stress and were not included in our analysis, since mild drought stress did not clearly distinguish the lines with high yield had potential from drought tolerant lines (Verulkar et al., 2010). Thus, the irrigated control category has 54 trials, 28 trials were classified as moderately stressed and 31 trials were categorized as severely stressed.

The genotypes screened included: (i) advanced breeding lines of medium (100–120 days) duration generated using crosses of popular high yielding varieties with a diverse array of donors for drought tolerance, (ii) popular varieties as check, and (iii) traditional drought tolerant landraces. Most of the popular checks and landraces used in this study were medium duration, similar to developed breeding lines. However, some checks like Swarna are long duration in maturity and were planted 10 days earlier to synchronize flowering of breeding lines in the reproductive-stage. Each year, newly nominated lines from each participating center and IRRI were jointly evaluated in an augmented-design in observational yield trials (OYTs), both in irrigated control conditions and under severe reproductive-stage stress, along with the checks. Promising lines from OYT screens were promoted to the AYTs (Verulkar et al., 2010). The criteria for selection of genotypes was based on grain yield advantage of the genotype shown under irrigated control as well as under moderate and severe drought stress conditions. The details of the number of genotypes and their connectivity across years are presented in Table 3.

**Table 3:** Details of the number of genotypes and their connectivity across years under irrigated control and the two stress levels in AYT trials.

No. of years tested	No. of genotypes		
	Irrigated control	Moderate stress	Severe stress
1	242	214	214
2	72	128	58
3	30	69	19
4	15	64	14
5	3	5	14
6	1	12	3
7	1	8	
8		9	1
9	1		1
10	1		
Total no. of genotypes	366	325	324

### 2.2 Dissecting genetic and non-genetic trends

The analysis was based on the model dissecting genetic trends from non-genetic trends in a two-stage analysis provided by Piepho et al. (2014). Genetic trends are due to breeding efforts, which can be assessed based on the year a genotype first entered the trials (birth). Non-genetic trends are due to agronomic efforts or the external factors that equally affect all genotypes (trial year), and they can be assessed based on calendar years.

The trials (site × year combinations) were analyzed using a two-stage analysis. In the first stage, data from individual trials was analyzed using a mixed model that considered genotypes as fixed and replicate and block effects as random to calculate the adjusted means for the genotypes in the *e*th trial with an estimated variance-covariance matrix $V_{e}$. If the adjusted means are sorted by trials (by site, year) and then by genotypes within each trial, *V* is of the block-diagonal form

$$V=\;{⊕}_{e=1}^{m}V_{e}=\left(\begin{array}{cccc} V_{1} & 0 & \ldots & 0\\ 0 & V_{2} & \ldots & 0\\ \vdots & \vdots & \ddots \; & \vdots \\ 0 & 0 & \ldots & V_{m} \end{array}\right)$$

where ⊕ is the direct sum operator, *m* is the number of trials and *V_e_* is the estimated variance-covariance matrix of adjusted means in the eth trial. A weighting matrix is given by $D\left(V_{e}^{-1}\right)$, a diagonal matrix with diagonal elements equal to those of $V_{e}^{-1}$ (Smith et al., 2001 and 2005).

In the second stage, for a given stress level, the basic model for the adjusted means is given by

(1) $$\hat{Y_{ijk}}=\mu +\;g_{i}+y_{k}+{\left(gy\right)}_{ik}+l_{j}+({ly)}_{jk}+({gl)}_{ij}+{\left(gyl\right)}_{ijk}+{\varepsilon \;}_{ijk}-----\;>\;$$

where $\hat{Y_{ijk}}$ is the adjusted mean of the ith genotype in the jth site and kth year, μ is the overall mean, $g_{i\;}$ is the main effect of the ith genotype, $l_{j}$ is the main effect of the jth site, $y_{k\;}$ is the main effect of the kth year, $({ly)}_{jk}$ is the interaction of the jth site with the kth year, ${\left(gy\right)}_{ik}$ is the interaction of the ith genotype with the kth year, ${\left(gl\right)}_{ij}$ is the interaction of the ith genotype with the jth site, ${\left(gyl\right)}_{ijk}$ is the interaction of the ith genotype with the jth site in the kth year and $\varepsilon_{ijk}$ is the error. The regression terms for the time trends for the main effects of the genotype effect $g_{i\;}$ and the year effect $y_{k}$ are then incorporated into the model. The genotypic main effect $g_{i\;}$ is modeled as a function of the year of first testing ($r_{i}$) for the ith variety

$$g_{i}=\;\beta r_{i}+\;H_{i}$$

where , β is the fixed regression coefficient for the “genetic” trend and $H_{i}$ is the random deviation of from the genetic trend line, $H_{i}∼N\left(0,\;\sigma_{H}^{2}\right).$ The year main effect can be modeled using the calendar year *t_k_* of the *k*th year as

$$y_{k}=\;\gamma t_{k}+\;Z_{k}$$

where γ is the fixed regression coefficient for the “agronomic” trend and $Z_{k}$ is the random deviation from the agronomic trend line, $Z_{k}∼N\left(0,\;\sigma_{Z}^{2}\right)\;.$ The model was fitted using PROC HPMIXED of SAS (SAS 9.3). Damesa et al. (2017) provided a SAS macro to calculate the weights proposed by Smith et al. (2001) for the two-stage analysis. Percentage change in genetic gain per year for each stress level due to the genetic and non-genetic causes were estimated as;

$$\frac{reg.\;coeff}{\mu +(reg.\;coeff\;x\;start\;year\;mean)}\;\text{x}\;100$$

where μ is the overall intercept (the grand mean across all the groups with no predictors), regression coefficient is β and γ for genetic and non-genetic causes, respectively and start year is start of the period over which gain was assessed.

### 2.3 Head-to-head trials

Head-to-head trials are a method of field demonstration initiated in the STRASA project, which allow the farmers to assess the yield performance of new variety with the already existing one under variable growing conditions in a rainfed system. The STRVs developed under STRASA were evaluated in head-to-head trials in farmers’ fields in the eastern Indian state of Odisha under two ecosystems: (i) the lowland and (ii) mid -and -shallow lowland. In the current study, within the lowland ecosystem, in addition to the topography, areas prone to submergence are referred to lowland, areas prone to occurrence of both drought and/or submergence in the same or different seasons have been referred to mid lowland and areas prone to frequent drought stress are referred to shallow lowland.

In the head-to-head trials, the pair of varieties to be compared were grown in the same field (half of the field for each variety) or in two adjacent fields with similar conditions. The head-to-head trials were conducted using the farmers management practices in the particular locations to assess the extent of yield gain achieved on the farmers’ fields (Dar et al., 2018). The data was analyzed separately for the lowland and shallow-and-medium lowland ecosystems.

The data was subjected to analysis of variance with fixed effects for variety, and random effects for districts and farmers nested within districts. Yield of the ith variety from the kth farmer in the jth district is modelled by

(2) $$y_{ijk}=\mu +t_{i}+e_{j}+f_{jk}+{\left(te\right)}_{ij}+\varepsilon_{ijk\;}$$

where, μ is the overall mean, $t_{i}$ is the effect of the ith variety, $e_{j}$ is the effect of the jth district, $f_{jk}$ is the effect of the kth farmer in the jth district, ${te}_{ij}$ is the effect of the district by variety interaction, $\varepsilon_{ijk}$ is the error. Heterogeneity of error variances among environments was investigated by examining a plot of the standardized residuals against the fitted response for both models (Raman et al., 2012; Hu et al., 2013). The models with homogeneous and heterogeneous error variances for districts were compared. The best model was chosen depending on the AIC values. In each ecosystem, the individual varieties were then classified into (i) STRVs and (ii) farmer’s variety. The model was rerun to compare the performances of the two categories.

## 3 Results

### 3.1 Single trial analysis of AYT

**Fig. 1:**
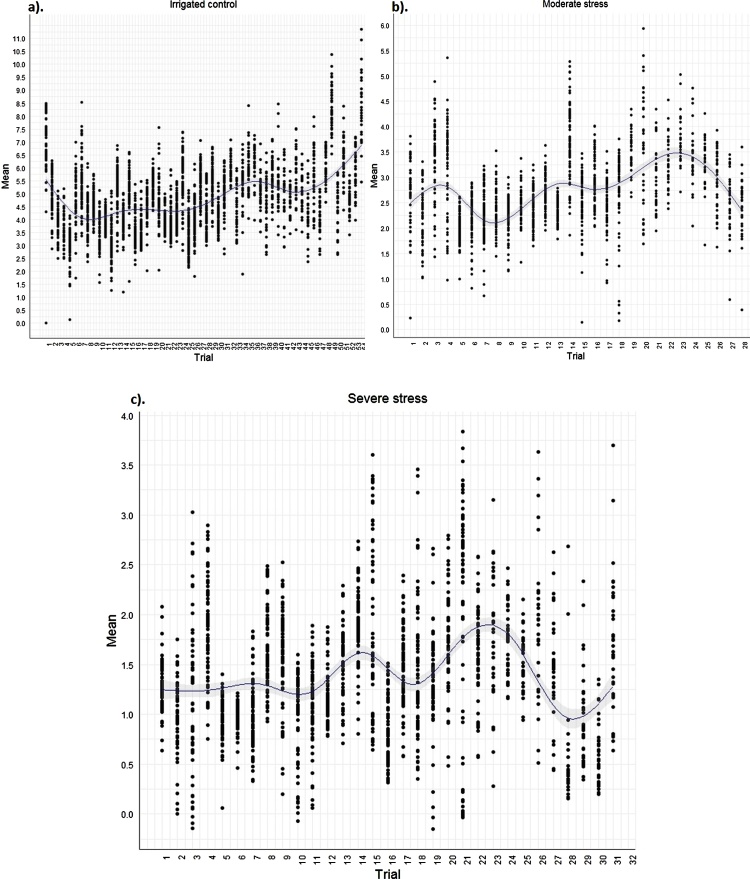
Adjusted mean yields from single trial-wise analysis of AYT.

The mean grain yield ranged from 2.5–8.0 t/ha in the irrigated control trials, 2–3.7 t/ha in the moderate drought stress trials and 0.5–1.9 t/ha under severe drought stress trials. The trials with heritability less than 0.30 were excluded from the subsequent analysis (Johnson et al., 1955). This included 7 trials under control, 6 under moderate stress and 5 under severe stress. Heritability of the trials included in the analysis ranged from 0.36 to 0.99 under non-stress, 0.39 to 0.98 under moderate stress and 0.32 to 0.98 under severe stress (Table 2). A plot of the trial-wise adjusted means under the irrigated control and two stress levels is shown in Fig. 1(a, b & c).

### 3.2 Analysis across years and sites

**Fig. 2:**
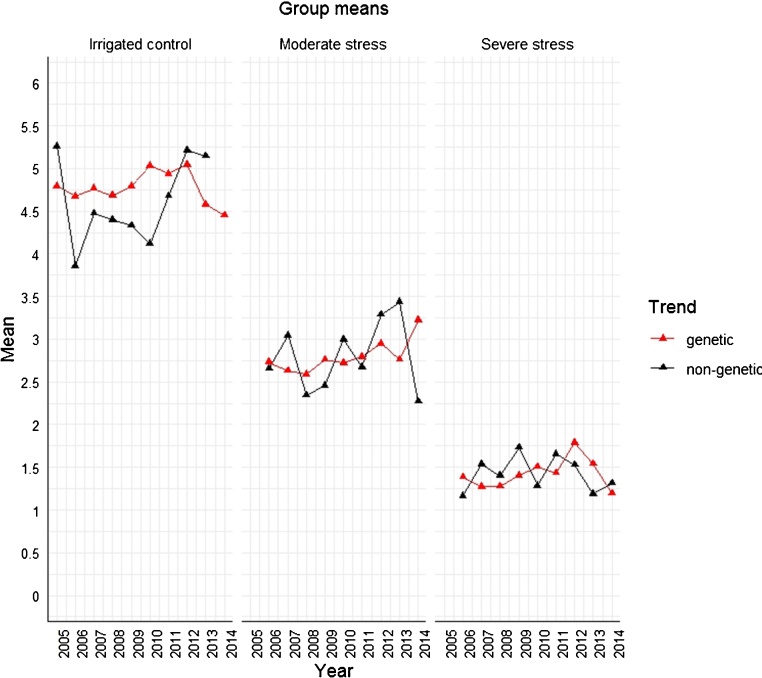
Marginal means of the variety groups based on the year of first testing (r_i_) and the calendar year (t_k_).

Comparing the regression estimates for the genetic and the non-genetic trends, it is observed that the genetic trend was positive with an increase in yield at the rate of 0.034 t/ha (confidence interval, CI: 0.007, 0.060) per year under irrigated control where the numbers in parenthesis indicate the lower and upper confidence limits at α = 0.05. The trend under moderate stress was 0.025 t/ha (CI: -0.004, 0.053) and 0.027 t/ha (CI: 0.004, 0.053) under severe stress. This can be interpreted as an increase of about 0.68 % p.a. under non-stress, 0.87 % p.a. under moderate stress and 1.9 % p.a. under severe stress (Table 4). The non-genetic trend was positive with an increase of about 0.122 t/ha (CI: 0.005, 0.239) under irrigated control and 0.05 t/ha (CI: -0.062, 0.135) in moderate stress, respectively. Under severe stress, a negative trend was found with a yield decrease of 0.021 t/ha (CI: -0.083, 0.051) (Table 4). The percentage increase was about 2.3 % p.a. under non-stress, 1.89 % p.a. under moderate stress and 1.5 % p.a. decrease under severe stress. A plot of marginal means of the variety groups based on the year of first testing (r_i_) and the calendar year (t_k_) for the irrigated control and two stress levels are presented in Fig. 2. In all the conditions, the year × site interaction variance was relatively large compared to the variances of all effects involving genotype. The genotype × year and genotype × site interaction variances were small compared to the genotype × site × year interaction variance (Table 5).

**Table 4:** Estimates of regression coefficients for the genetic and non-genetic trends and estimates of their group means from the analysis across sites and years for the AYT trials under the irrigated control, moderate and severe drought stress.

	Irrigated control		Moderate stress		Severe stress	
Regression coefficient	est	std. err	est	std. err	est	std. err
β	0.034	0.014	0.025	0.015	0.027	0.012
γ	0.122	0.059	0.055	0.055	−0.021	0.032
Group means						
Year	r_i_	t_k_	r_i_	t_k_	r_i_	t_k_
2005	4.793	5.258				
2006	4.673	3.861	2.730	2.656	1.384	1.164
2007	4.765	4.476	2.630	3.042	1.270	1.537
2008	4.682	4.398	2.593	2.343	1.279	1.401
2009	4.795	4.331	2.764	2.458	1.404	1.732
2010	5.033	4.119	2.724	2.999	1.507	1.284
2011	4.939	4.681	2.796	2.672	1.434	1.653
2012	5.047	5.212	2.948	3.290	1.787	1.529
2013	4.578	5.148	2.765	3.436	1.544	1.188
2014	4.450	6.271	3.221	2.272	1.195	1.315
average	4.775	4.775	2.797	2.797	1.422	1.422
% gain#	0.68	2. 3	0.87	1.9	1.9	−1.5

Note: est – estimate; std. err – standard error. r_i_ – year of first testing – genetic trend ; t_k_ – trial year – non genetic trend; β is the fixed regression coefficient for the “genetic” trend r_i_ ; γ is the fixed regression coefficient for the “agronomic” trend t_k._

# Percentage change in genetic gain per year for each stress level due to the genetic and non-genetic causes were estimated using the formula explained under subheading dissecting genetic and non-genetic trends in materials and methods section.

**Table 5:** REML parameters from the analysis across sites and years for the AYT trials under irrigated control and the two stress levels.

Cov Parm	Irrigated control			Moderate stress			Severe stress		
	Estimate	Standard Error	p-value	Estimate	Standard Error	p-value	Estimate	Standard Error	p-value
Year	0.0841	0.1454	0.281	0.112	0.086	0.096	0.004	0.034	0.453
Gen	0.080	0.015	<.0001	0.043	0.013	0.001	0.023	0.008	0.003
Site	0.031	0.141	0.413	0	.	.	0.034	0.041	0.206
year*site	0.918	0.231	<.0001	0.132	0.045	0.002	0.147	0.057	0.005
gen*year	0.000	0.010	0.500	0.000	0.011	0.500	0.003	0.007	0.343
gen*site	0.040	0.019	0.025	0.053	0.018	0.002	0.021	0.011	0.025
gen*year*site	0.339	0.028	<.0001	0.162	0.019	<.0001	0.161	0.013	<.0001
Residual	1	0		1	0		1	0	

Note: gen – genotype; Cov Parm-covariance parameter; REML: restricted maximum likelihood.

### 3.3 Analysis of head-to-head trials

**Fig. 3:**
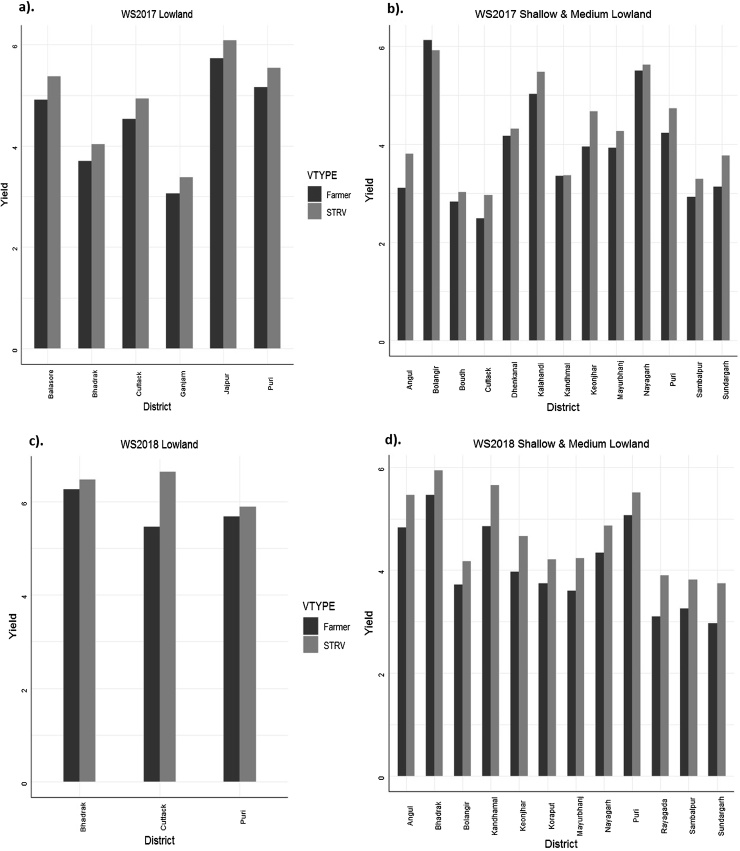
District-wise predicted means of the STRV and farmer varieties from head to head trials.

The varietal differences were non-significant (p < 0.05) in WS2017 and significant in WS2018 in lowland trials while it was highly significant in both years in the shallow and medium lowland trials based on *model 3* (Supplementary Table S2). Swarna -Sub1 had produced the highest yield in the lowland ecosystem in both years while DRR dhan 44 had yielded highest in the shallow and medium lowland situation (Table 6). The STRVs outperformed the currently grown varieties on farmers’ fields in all situations in both years (Fig. 3. a,b,c & d).

**Table 6:** Estimated variety means from the head-to-head trials at Odisha under lowland and shallow & medium lowland conditions.

Variety type	Lowland				Shallow & medium lowland			
	WS2017		WS2018		WS2017		WS2018	
	Mean	std err	Mean	std err	Mean	std err	Mean	std err
Farmer	4.540	0.410	5.818	0.248	3.788	0.266	4.154	0.217
STRV	4.855	0.410	6.328	0.248	4.240	0.266	4.708	0.217
STRV								
Bina dhan 11					4.193	0.267	4.621	0.233
Swarna Sub 1	4.873	0.438	6.2157	0.1741				
CR 1009 Sub -1	4.846	0.482	6.5717	0.1924				
DRR dhan 42					4.203	0.270		
DRR dhan 44					4.522	0.276	4.777	0.243
Sahbhagi dhan					4.068	0.286	4.593	0.246
Farmer variety								
CR 1009	3.795	0.592	6.2697	0.2094				
Khandagiri					3.417	0.423	4.036	0.326
Lalat					3.732	0.270	3.978	0.239
MTU 1001	4.466	0.497			4.253	0.308	4.313	0.258
MTU 1010					3.891	0.312	4.138	0.250
Naveen					3.758	0.267	4.067	0.252
Pooja	4.430	0.478	5.3043	0.1952				
Sarala	4.304	0.789	5.7207	0.3094				
Swarna	4.693	0.439	6.119	0.1833	3.985	0.386	4.855	0.317
Swarna Shreya							4.870	0.359

**Note:** STRV-stress tolerant rice varieties.

## 4 Discussion

The increase in frequency and intensity of drought due to climate change necessitates the use of varieties that possess (i) drought resistance together with high yield under favorable conditions (Serraj et al., 2011), (ii) appropriate growth duration based on toposequence of the land, (iii) resistance to biotic stresses, (iv) improved grain quality and (v) other traits of economic importance. These goals can be achieved with large-scale breeding programs targeted to achieve a continuous cycle of varietal improvement, wherein older varieties are replaced with new varieties that can meet farmers’ needs and thus deliver genetic gains.

Genetic gain assessment is vital for evaluating genetic improvement, selection efficiency and for designing future breeding strategies (Fufa et al., 2005). Breeding programs designed for the rainfed ecosystem targeting the developing world will need to deliver higher rates of genetic gain to cope with the 21 st century challenges of 50 %–60 % greater demands for food commodities, climate change and natural resource constraints (Genetic Gains Working Group, 2016). IRRI, in collaboration with national research partners under STRASA, initiated efforts for developing drought-tolerant rice varieties suitable for rainfed lowland drought-prone areas of India. Many popular high yielding rice varieties are highly susceptible to drought, hence IRRI implemented a breeding strategy for the development and evaluation of breeding lines in on-station trials under control as well as moderate stress and severe reproductive stage drought stress (Kumar et al., 2007; Venuprasad et al., 2007; Verulkar et al., 2010; Mandal et al., 2010) with the aim to combine drought tolerance with high yield potential. Screening under moderate and severe drought stress exposed the breeding lines to a range of severity of reproductive stage drought stresses likely to prevail in the rainfed regions. The efforts led to development of several high yielding drought tolerant lines (Kumar et al., 2014). Genetic gain assessment of grain yield in the present study clearly indicates that the efforts have been successful in not only increasing the yield under moderate and severe reproductive stage drought stress but also under irrigated control. Further, the evaluation of breeding lines at segregating generations as well as at the advanced stage under the three situations ensured obtaining genetic gains under all the three conditions. The genetic gain of 0.68 % under control, 0.87 % under moderate stress and 1.9 % under severe reproductive stage drought stress demonstrates the success of our breeding strategy implemented between 2004–2014. Earlier, Xangsayasane et al., 2013 reported that screening of lines under intermittent drought in the dry season and terminal drought in the wet season provided the magnitude of drought severity that was appropriate identifying promising drought tolerant lines. Based on drought response index (DRI), genotypes shown to be drought tolerant were consistent in performance in intermittent and terminal drought screening (Xangsayasane et al., 2013). Promising genotypes with good yield potential under irrigated control as well as under moderate and severe drought stress had been previously selected based on drought yield index (DYI) and mean yield index (MYI) in rainfed drought prone locations in Eastern India (Raman et al., 2012).

The genetic gains achieved by the breeding programs in on-station trials between 2004–2014 were also observed in the on-farm trials conducted during 2017−2018. The performance of some of the developed breeding lines released as varieties such as Swarna -Sub 1 for lowland area, Sahbhagi dhan, DRR dhan 42, DRR dhan 44, Bina dhan 11 for mid and shallow lowland areas were evaluated in comparison with the varieties farmers were growing (Swarna, CR1009, Khandagiri, Lalat, MTU1010, Naveen, Pooja, Sarala) in the rainfed regions in on-farm trials. Swarna-Sub1 showed an average yield advantage of 0.315 and 0.510 t/ha in 2017 and 2018 respectively over mean performance of varieties farmers were growing in the lowland including Swarna. In shallow and medium lowland, the drought tolerant varieties such as Sahbhagi dhan showed an average yield advantage of 0.452 and 0.554 t/ha in 2017 and 2018 respectively over the mean performance of varieties under cultivation by farmers. These findings are in agreement with the Xangsayasane et al., 2014 who have earlier reported that drought tolerance determined in either intermittent or terminal drought in on-station trials was effective in predicting on-farm yield.

For long-term genetic improvement of yield, evaluating the genetic variation present in the germplasm is a pre-requisite (Allard, 1960; Falconer and Mackay, 2004), as availability of adequate variability provides a range of choices from which selections can be made. Genetic variability for yield under drought stress in rainfed lowland rice in both dry and wet seasons have been documented (Venuprasad et al., 2007; Kumar et al., 2007; Verulkar et al., 2010; Gouda et al., 2012; Saikumar et al., 2016; Shaibu et al., 2017). The genotypic variance component observed for yield under irrigated control and two stress levels in the present study was larger than that of the genotype × year and the genotype × site variance components suggesting enough genetic variability in the breeding material used.

When assessing cultivar performance and genetic gains at multiple sites and across years, breeding and varietal selection may not be the only major factors responsible the increase in grain yields (Ahrends et al., 2018; Muralidharan et al., 2019). Long-term genetic gains for yield under a range of drought situations can be achieved by combining genetics and crop management (Duvick et al., 2004; Cooper et al., 2014). This is because responses to drought stress are complex and also involve climatic, soil and agronomic factors and therefore predictions of phenotypic response should be made as functions of genetic and environment factors. Selection of superior genotypes depends on the quality of these predictions (Sadras et al., 2013; Bustos-Korts et al., 2016). In the present study, regression estimates show that in all stress levels, yield increase is mainly caused by new varieties rather than by the agronomic factors. The contribution of the environmental factors under irrigated control and moderate stress were positive while it was negative in the case of severe stress trials. This could be attributed to the drought stress in these environments. Assessing the size of the contributions of genetic, environmental and management factors can have implications for on-farm planning, testing breeding strategies and allocation of resources for yield improvement (Anderson, 2010).

Determination of genetic progress in grain yield over a period has been extensively studied in other grain crops such as wheat and maize but has been limited in the case of rice. Peng et al. (2000) determined the trend in rice yield of cultivars developed by IRRI between 1966 and 1996. Their study indicated a gain of 1% per year. Muralidharan et al. (2002) analysed the grain yield performance of rice genotypes developed and tested since 1976–1997 in the METs performed worldwide under different ecosystems in the international rice improvement trials and concluded that there was no evidence for either a genetic gain or loss in grain yields of genotypes developed for any of the ecosystems. Further, Muralidharan et al. (2019) observed a significant yield gap between the projection of human population and national rice grain production by estimating the genetic gain for yields in genotypes tested in 11 rice ecosystems from 1995 to 2013 in India. Integrated genomic technologies and policy interventions is needed to seal this yield gap and to ensure rice availability to fulfill the demands of growing population.

Bastasa et al. (2015) determined the annual genetic gain in rice yield based on 28 cultivars representing different years of varietal release over the time period 1966–2013 and reported an annual yield gain of 1% on the basis of yield of IR64. Saito (2018) assessed the trend in grain yield and its associated traits of rice varieties released during the last decades in sub-Saharan Africa. The study showed considerable genetic gains in rainfed systems and high yielding lowland varieties released between 1986 and 2013 had around 20 % higher yield than IR64.

In studies assessing the genetic gain for grain yield in different crops, yield is regressed against the year of release to monitor the gains in released varieties over time as also reported in other crops (Sayre et al., 1997; Lopes et al., 2012; Masuka et al., 2017). An alternate method of assessing long-term genetic gain based on MET data is to evaluate cultivar performance trials sequentially over a period of time in the collaborating test sites such as in the present study. Grain yield regressed against the year of testing and the performance of the test varieties are compared with that of common checks with the main objective of variety release (Trethowan et al., 2002; Tadesse et al., 2013).

Among the drought tolerant varieties developed and released following strategy of evaluation of breeding lines for grain yield under irrigated control and reproductive stress, Sahbhagi dhan, was the first variety released in 2009 as a result of the collaboration between IRRI and different Indian institutions for drought-prone shallow lowland ecosystems of India (Basu et al., 2017) as well as for bunded uplands. Sahbhagi dhan had a comparatively stable performance and adaptable plant types for shallow rainfed lowland drought-prone ecosystems (Verulkar et al., 2010; Kumar et al., 2012; Raman et al., 2012) and has shown a yield advantage of 0.8–1.0 t/ha over other varieties under drought conditions (Yamano et al., 2013) where the yield of irrigated control varieties such as IR 64 collapsed (Dar et al., 2014). DRR dhan 44, released in the year 2014 for cultivation under irrigated conditions, is characterized by high yield under water limited conditions. Other drought-tolerant varieties released for commercial cultivation in India include DRR dhan 42, 43, 50 and CR dhan 204, 205, 801 and 802.

In lowland, Swarna- Sub1 performed better than any other variety closely followed by CR 1990 Sub 1. In mid lowland, in lower bunds, Bina dhan 11 performed exceedingly well whereas in mid lowland upper bunds as well as in shallow lowland DRR dhan 44 performed better than other varieties including the most presently cultivated variety MTU1010 (Table 6).

## 5 Conclusion

This study documents the significant genetic gain for grain yield of a breeding program targeting rainfed lowland rice in India that was based on direct selection for grain yield under both irrigated control and drought conditions. The study utilized extensive multi-season evaluation in target environments under irrigated control, moderate drought stress and severe drought stress between 2004–2014 with number of popular varieties as checks to enable accurate estimation of the genetic gain. The yield improvement of the newly developed stress-tolerant varieties over the best currently grown varieties was also demonstrated on farmers’ fields. The developed STRVs have potential to protect farmers from crop losses against an increasing impact of extreme droughts under climate change. The results of this study shall assist governments and policy makers to replace the currently grown decades-old varieties from the seed chain and emphasize larger efforts for seed multiplication and dissemination of the newly developed stress-tolerant varieties in coordination with various stakeholders. The findings of the study shall also encourage researchers to plan for effective evaluation of the breeding programs products with aim to assess the success of the breeding programs in terms of genetic gains achieved.

## Availability of data and materials

The datasets supporting the conclusions of this article are provided within the article.

## Authors’ contribution

**AK**: was involved in design of overall experiment, drafting and critical revision of MS; **AR:** was involved in experimental analysis, interpretation of data, drafting and revising the manuscript; **SY:** was involved in evaluating the lines at IRRI and revising the manuscript; **SBV:** conducted trials and recorded measurements at IGKV Raipur; **NPM:** performed trials at Hazaribagh, **ONS** and **PS**: conducted the trials and recorded measurements at NRRI Cuttack, **TR** and **JB**: conducted the trial and recorded observations at IIRR-Hyderabad; **JLD:** performed the trials at NDUAT-Ayodhya; **SPD**: conducted the trials at ICAR-Tripura, **SKS:** conducted the trial at BHU-Varanasi; **SPS:** conducted the trial and recorded data at BAU-Sabour; **SK:** conducted the trial at ICAR-Patna; **AJ**: conducted trials and recorded observations at Barwale foundation, Hyderabad; **RC** and **SR:** conducted trials and recorded observations at TNAU Coimbatore; **HES** and **SH**: conducted the trials and recorded data at UAS-Bangalore, **PS**: conducted the trials and recorded the required data at ANGRAU-Guntur, **VC, JR** and **SN**: were involved in multiplication of seeds conducted the trials at IRRI-SAH, Hyderabad, and distributed the multiplied seeds to partners at various locations; **SN, MD** and **MH:** had monitored and evaluated the trials at various locations in India; and **AH**: performed screening of donors for drought tolerance at IRRI under the STRASA project. **HPP**: provided suggestions regarding the experiments and statistical models and analysis and also helped in editing of the MS. All authors have read and approved the final manuscript.

## Ethics approval and consent to participate

Not applicable.

## Consent for publication

Not applicable.

## Declaration of Competing Interest

The authors declare that they have no known competing financial interests or personal relationships that could have appeared to influence the work reported in this paper.
